# Demystifying Excess Immune Response in COVID-19 to Reposition an Orphan Drug for Down-Regulation of NF-κB: A Systematic Review

**DOI:** 10.3390/v13030378

**Published:** 2021-02-27

**Authors:** Apparao Peddapalli, Manish Gehani, Arunasree M. Kalle, Siva R. Peddapalli, Angela E. Peter, Shashwat Sharad

**Affiliations:** 1 Department of Microbiology, King George Hospital, Visakhapatnam 531011, Andhra Pradesh, India; vatavaapparao@gmail.com; 2 Department of Biological Sciences, Birla Institute of Technology and Science, Pilani-Hyderabad Campus, Hyderabad 500078, Telangana, India; dr.manishgehani@gmail.com; 3 Department of Animal Biology, School of Life Sciences, University of Hyderabad, Hyderabad 500046, Telangana, India; arunasreemk@uohyd.ac.in; 4 Department of Biological Sciences-Biotechnology, Florida Institute of Technology, Melbourne, FL 32901, USA; peddapalli.rasagnya@gmail.com; 5 Department of Biotechnology, College of Science & Technology, Andhra University, Visakhapatnam 530003, Andhra Pradesh, India; angelapeter.728@gmail.com; 6 Center for Prostate Disease Research, John P. Murtha Cancer Center Research Program, Department of Surgery, Uniformed Services University of the Health Sciences and the Walter Reed National Military Medical Center, Bethesda, MD 20817, USA; 7 Henry M. Jackson Foundation for the Advancement of Military Medicine, Bethesda, MD 20817, USA

**Keywords:** COVID-19, cytokine storm, SARS-CoV-2, repositioning, histamine-conjugated-normal human immunoglobulin, excess immune response, hyper-inflammation

## Abstract

The immunological findings from autopsies, biopsies, and various studies in COVID-19 patients show that the major cause of morbidity and mortality in COVID-19 is excess immune response resulting in hyper-inflammation. With the objective to review various mechanisms of excess immune response in adult COVID-19 patients, Pubmed was searched for free full articles not related to therapeutics or co-morbid sub-groups, published in English until 27 October 2020, irrespective of type of article, country, or region. Joanna Briggs Institute’s design-specific checklists were used to assess the risk of bias. Out of 122 records screened for eligibility, 42 articles were included in the final review. The review found that eventually, most mechanisms result in cytokine excess and up-regulation of Nuclear Factor-κB (NF-κB) signaling as a common pathway of excess immune response. Molecules blocking NF-κB or targeting downstream effectors like Tumour Necrosis Factor α (TNFα) are either undergoing clinical trials or lack specificity and cause unwanted side effects. Neutralization of upstream histamine by histamine-conjugated normal human immunoglobulin has been demonstrated to inhibit the nuclear translocation of NF-κB, thereby preventing the release of pro-inflammatory cytokines Interleukin (IL) 1β, TNF-α, and IL-6 and IL-10 in a safer manner. The authors recommend repositioning it in COVID-19.

## 1. Introduction

Severe acute respiratory syndrome coronavirus 2 (SARS-CoV-2) belongs to the family Coronaviridae of the Nidovirales order. Its genome is a positive-sense single-stranded ribonucleic acid (RNA). It enters the body with the help of its spike protein (S protein) by infecting airway epithelium cells, alveolar epithelial cells, vascular endothelial cells, and macrophages in the lung through human angiotensin-converting enzyme 2 (ACE2) receptors [1,2,3]. The virus enters the cells mainly through endocytosis, with the help of phosphatidylinositol 3-phosphate 5-kinase (PIKfyve), two-pore channels 2 (TPC2), and cathepsin L [3,4]. Transmembrane serine protease 2 (TMPRSS2) helps in priming the S protein [5]. The virus entry reduces ACE2 expression, which results in a dysfunction of the renin–angiotensin system (RAS). This influences blood pressure and fluid–electrolyte balance while causing inflammation and enhancing vascular permeability in the airways [6]. After the entry, it starts replicating actively at the site of infection. It is also reported to directly invade other organs and proliferate in the gut with the help of ACE2 receptors in these organs [7,8,9,10,11,12,13]. Hamming et al. studied the expression of ACE2 in various human organs and found that it was expressed on lung alveolar epithelial cells; enterocytes of the small intestine; and arterial and venous endothelial cells and arterial smooth muscle cells in oral and nasal mucosa, nasopharynx, lung, stomach, small intestine, colon, skin, lymph nodes, thymus, bone marrow, spleen, liver, kidney, and brain [14]. The clearance of viral RNA in stools of patients is more delayed as compared to the respiratory system [13]. 

### 1.1. Normal Response of the Human Immune System to SARS-CoV-2 Infection

**Figure 1:**
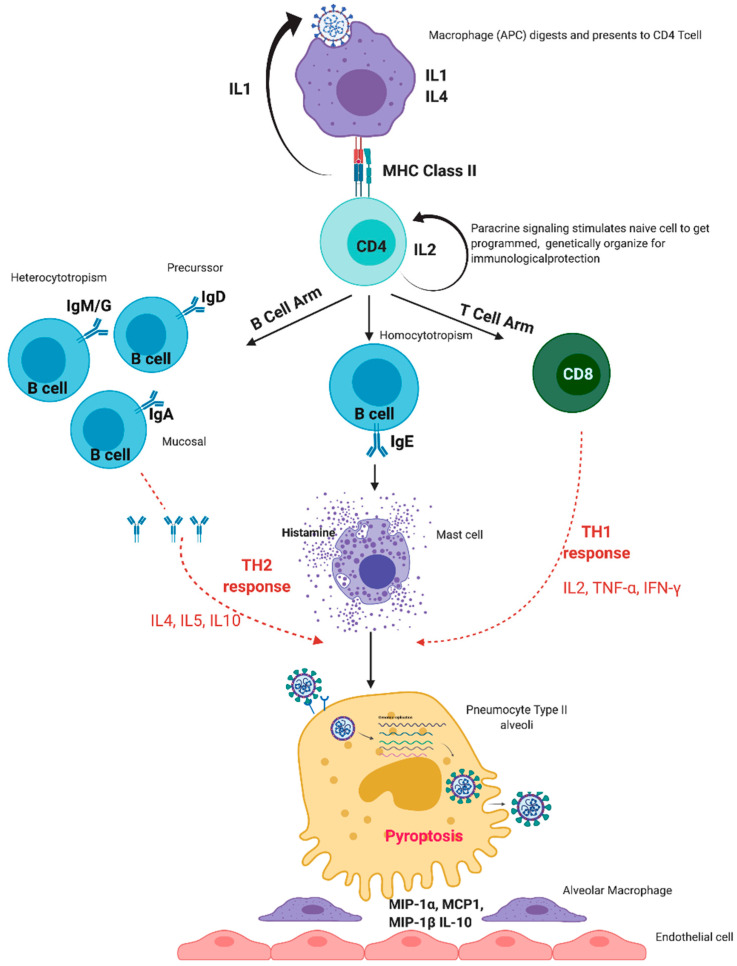
Normal immune response of the human immune system to SARS-CoV-2 infection. MIP: Macrophage inflammatory protein-1 alpha, MCP: monocyte chemoattractant protein.

Exposure to the virus results in variable severity of disease ranging from asymptomatic or mild disease to severe disease. In a normal scenario, the human immune system elicits a regulated immune response to SARS-CoV-2 infection. The release of the virus causes the host cells to undergo a phenomenon known as pyroptosis (Figure 1) [6], which releases molecular patterns and triggers innate immunity. The innate immunity causes the creation of barrier, secretion of mucus, and shredding of epithelial layer containing virus. Neutrophils engulf the enemy by phagocytosis and produce enzymes to kill it [15]. Natural killer (NK) cells cause apoptosis of rogue self-cells containing the pathogens [16]. Expression of pro-inflammatory genes is induced with the help of factors like Nuclear Factor-κB (NF-κB) [17]; as a result, pro-inflammatory cytokines and chemokines are released, which attract monocytes, macrophages, and T cells to the site of the insult [6]. Type I interferons (IFN) IFN-α, and IFN-β block viral replication and augment antiviral effector mechanisms. SARS-CoV-1 and likely the homologous SARS-CoV-2 expresses proteins that inhibit type I IFN production [18]. This delays the antiviral response and facilitates rapid viral replication and extensive virus-induced direct cytopathic effects in the early stages of disease [18].

Along with triggering of the innate system, the adaptive immune system is also started. Timing of adaptive immune response is vital for efficient viral clearance by innate immunity [19]. Macrophages or dendritic cells engulf the viruses and function as antigen-presenting cells. With the help of Interleukin (IL)-1, these antigen-presenting cells present small epitope antigens to the immature cluster of differentiation (CD)4+T cells by major histocompatibility complex (MHC) class 2 receptors in the presence of a co-signal molecule called CD27 and form mature helper T cells [20]. IL-2 acts in an autocrine manner and determines which foreign particles are extracellular and need an antibody response, and which are intracellular and need T-cell response [20,21]. 

Depending upon the pattern of cytokine release, T helper (Th) cells are differentiated into either Th1 or Th2 effector cells, and histamine regulates the balance of both [22]. Th1 type helper cells promote cell-mediated immunity by activating macrophages, while Th2 type helper cells activate B-cell proliferation [21]. Similarly, antigen is presented to immature CD8+T cells through MHC 1 to form mature cytotoxic T cells [20], which break open the infected cells, and the foreign organisms are killed once they come out of the broken cell [23].

The T cells and B cells meet in the secondary lymphoid organs and exchange information, due to which naïve B cells are activated to become effector B cells, which secrete disease-antigen-specific antibodies or immunoglobulins (Ig) of five types—IgD, IgA, IgE, IgG, and IgM. IgD aids the activation of naïve B-cells. IgA acts locally on the site of insult. Initially IgM and later IgG target foreign particles by hetero-cytotrophism [24]. IgE targets the body’s own mast cells by homo-cytotrophism, causing degranulation and histamine release [24]. 

Studies have found that histamine plays a key role in coronavirus disease 2019 (COVID-19) due to its immunomodulatory actions on mast cell histamine–cytokine cross-talk [25]. Histamine acts through four receptors H1, H2, H3, and H4. The H1 receptor is ubiquitous and is activated through guanine nucleotide-binding protein (G) αq; H2 is highly expressed in B-cells, T-cells, gastric parietal cells and is coupled to Gαs; H3 is exclusively expressed in neurons and is coupled to Gαi/o; and H4 is found on immune cells, lung, central nervous system (CNS), spleen and is coupled to Gi/o [26]. Histamine activates other mediator biomolecules, such as serotonin, arachidonic acid metabolites, platelet-activating-factor, complement mediator substances Complement (C) 3a and C5a, and cytokines. All these biomolecules, in return, can trigger histamine. In addition, the cumulative action of angiotensin II and vasopressin via Gq pathways contribute to inflammation [27,28,29,30]. 

The secreted histamine promotes vascular smooth muscle contraction and diapedesis of all immune cells, antibodies, and mediators into the site of insult [31]. In a normal scenario, these immune entities neutralize and kill all the foreign particles, and macrophages do scavenging. The body subdues what is not needed out of the immune molecules, following the law of conservation of mass. Histamine is neutralized by its biological antagonist adrenaline after the insult is resolved [32]. The serum also has histamine neutralizing ability, which takes care of the residual histamine. This histamine homeostasis is important for immune modulation and regulation of inflammation in the body [22].

### 1.2. Need for Reviewing Excess Immune Response of Human Immune System to SARS-CoV-2 Infection in this Pandemic Situation

Nearly 20% of COVID-19 patients develop serious complications due to excess immune response of the human immune system [33], resulting in pneumonitis, Acute Respiratory Distress Syndrome (ARDS), encephalopathy, hypercoagulability, pulmonary embolism, deep vein thrombosis, ischemic stroke, myocardial infarction, systemic arterial embolism, disseminated intravascular coagulation, virus-activated cytokine storm syndrome, fulminant myocarditis, septic shock, mimicry of vasculitis, endothelium damage, and multiple organ failure in humans [7,34,35,36,37,38]. Immune-mediated damage due to COVID-19 occurs in various organs. Lungs of patients of COVID-19 show marked alveolar inflammatory cell infiltrate, diffuse alveolar damage, formation of hyaline membranes, and diffuse thickening of the alveolar wall [35]. The spleen shows atrophy, and lymph nodes show necrosis with reduction of lymphocytes in lymphoid organs [35,36]. Older age, male gender, underlying co-morbidities, and secondary infections are reportedly associated with high fatality [39]. 

Many authors have described a multitude of mechanisms of the excess immune response of the human immune system to SARS-CoV-2, but to date, the complex and heterogeneous nature of hyper-inflammation in COVID-19 is not fully understood. The objective of this study was to review the reported extracellular and intracellular mechanisms in the patients showing excess immune response to SARS-CoV-2 infection in published articles, to suggest life-saving solutions in this pandemic, and to propose further research.

## 2. Materials and Methods

A systematic review was conducted to synthesize a narrative of mechanisms of excess immune response in patients of COVID-19. The protocol of conducting the review was registered in advance in the PROSPERO register with registration number CRD42020214230.

### 2.1. Search Strategy

MEDLINE database was searched through Pubmed using keyword:

(COVID-19 OR SARS-CoV-2 OR nCoV OR “Novel Corona”) AND (“excess immune response” OR “exaggerated immune response” OR “excessive immune response” OR “excess immunity” OR “exaggerated immunity” OR “excessive immunity” OR “hyperinflammation” OR “excess inflammation” OR “exaggerated inflammation” OR “excessive inflammation”) NOT (“clinical trials” OR “clinical trial” OR “trial”).

There was no restriction related to the type of article, country, or region, so as to include findings in a broad population studied at varied timings during the pandemic. Articles published in English, related to human species, and available as free full text were included. The last search was run on 27 October 2020. The search was not rerun prior to the final analysis and no unpublished studies were sought. 

As Pubmed provides clinical literature focused on human species, it suited the study question of the review regarding the excess immune response of the human immune system to SARS-CoV-2 virus. Although Pubmed yielded a sufficient number of articles that were made available free of cost during the pandemic, authors admit that searching only one portal and including articles published only in English may have introduced publication bias.

### 2.2. Selection of Studies

Studies describing mechanisms of the excess immune response of the human immune system to SARS-CoV-2 and the intra-cellular pathways among the reported mechanisms were studied. Study participants were COVID-19 patients eliciting excess immune response. Exposure was excess immune response in severe COVID-19 resulting in hyper-inflammation, as compared to normal immune response of the human immune system to SARS-CoV-2 infection. Two reviewers screened titles, abstracts, and full papers in an unblinded standardized manner and assessed the eligibility of retrieved articles based on the following criteria.

#### 2.2.1. Inclusion Criteria

1. The articles describing immunology in COVID-19 patients especially in the context of excess immune response;
2. Articles pertaining to adult population showing excess immune response in COVID-19.

#### 2.2.2. Exclusion Criteria

1. Articles related to drug repositioning, therapeutics, target drugs, therapies, treatments, and vaccines;
2. Articles focusing on a sub-group of patients suffering from a particular co-morbidity;
3. Articles with study design as clinical trial.

Articles utilizing a drug as a material to elicit a phenomenon or to describe immunological concepts or explaining drugs or pathways or therapeutic targets just as a suggestion were not excluded.

The reviewers were blinded to each other’s decisions. The screening process was validated, and disagreements were resolved by other co-authors of the paper and were recorded in excel sheets. 

### 2.3. Data Extraction

Extraction was done by one reviewer and was checked and verified by the other reviewer. Disagreements were resolved by mutual consensus. Data regarding study design, type of article, month of publication, participant demographics, mechanism of excess immune response, and mechanism of normal immune response were extracted from the studies in an Excel-based extraction sheet. Description of mechanisms of excess immune response in patients was compiled from the included articles and synthesized into a narrative. The extracted data were validated by all authors of this article.

### 2.4. Assessment of Risk of Bias and Quality of Studies

Quality assessment was done by two reviewers independently, and disagreements were resolved by consultation with each other. Assessment was done at the study level. Characteristics to be assessed depended upon the study design of the screened record. Joanna Briggs Institute’s (JBI) critical appraisal tools [40] for respective study design of articles were used. For reviews, the initial five criteria of the JBI checklist were considered critical, and the remaining six criteria were planned to be assessed only when the initial five were satisfied. For narrative reviews, which could not fulfill the initial five criteria of the JBI tool, the Scale for the quality Assessment of Narrative Review Articles (SANRA) tool [41] was used. For research papers with cross-sectional design and secondary data analysis, all criteria of the JBI tool for analytical cross-sectional studies were considered essential to be fulfilled for inclusion in the review. Similarly, for remaining articles like editorials, viewpoints, or comments, all criteria of the JBI tool for text and opinion were considered essential to be fulfilled for inclusion in the review. The data of only those studies were included in the final narrative synthesis, which fulfilled the criteria of quality assessment.

### 2.5. Strategy for Data Synthesis

A formal narrative synthesis was planned. A minimum of 10 studies was considered to be required for synthesis. Qualitative data aggregated in an Excel sheet was synthesized for presenting common mechanisms of excess immune response of COVID-19 patients and the intracellular pathways reported in the studies using the formal method "meta-study", including meta-theory, meta-method, and meta-data-analysis. Indication of interpretation of results was for deriving common mechanisms and exploring their common links or pathways at the intracellular level. Two researchers were involved in data synthesis, and the discrepancies were resolved by consultation among all co-authors.

### 2.6. Primary Outcome

Description of the mechanism of excess immune response of human immune system to SARS-CoV-2 and the intracellular pathways involved was the outcome of interest, where the immune response was measured based on the presence of immune cells, antibodies, and mediators of innate and/or acquired immunity found by any of the following analyses: immunological findings on autopsies, analysis of biomarkers, broncho-alveolar lavage fluid analysis, histopathological analysis, electron microscopy, and immunostaining. The measure of effect was the mortality rate due to various reported mechanisms for excess immune response.

## 3. Results

### 3.1. Flow of Studies through the Review Process

**Figure 2:**
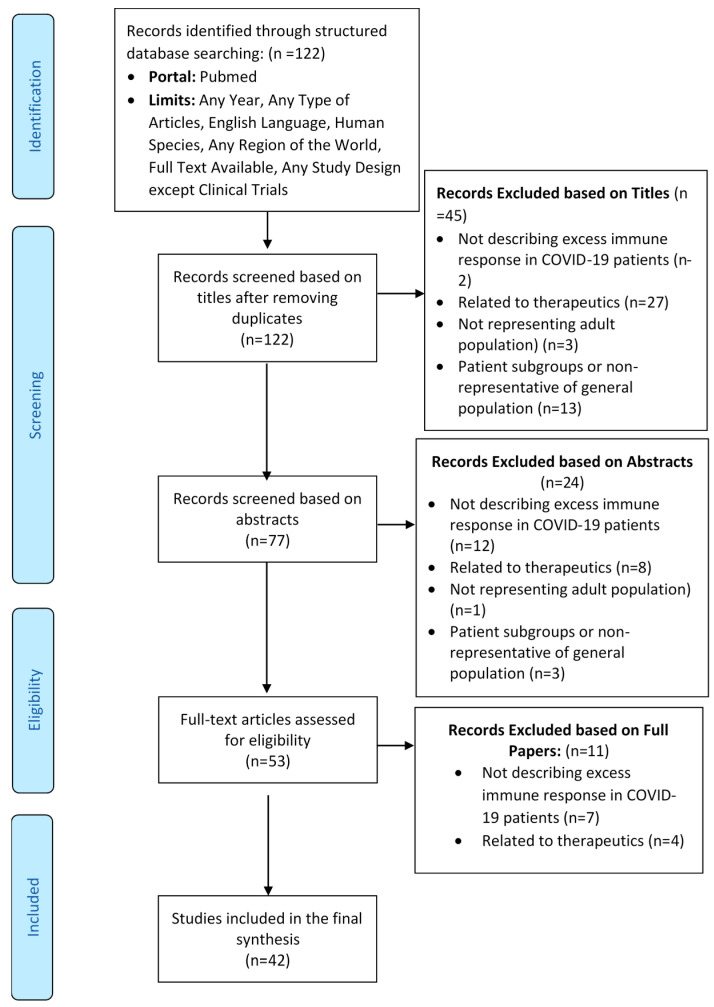
Preferred Reporting Items for Systematic Reviews and Meta-Analyses (PRISMA) diagram showing selection of studies for inclusion in the review.

Out of a total 122 records screened for eligibility, 42 articles were included in the final review. The flow of the studies through the review process is depicted in the Preferred Reporting Items for Systematic Reviews and Meta-Analyses (PRISMA) flow diagram provided below (Figure 2). All 42 included articles passed quality assessment as per their respective study design or article type. The details of the characteristics of the included studies are mentioned in Table 1. The studies included in the review were conducted in small geographies or limited patient populations and may not be generalizable over all of humankind.

Authors reported their findings based on different procedures like autopsies, analysis of biomarkers, broncho-alveolar lavage fluid analysis, histopathological analysis, electron microscopy, and immunostaining. 

### 3.2. Mortality Rate due to Excess Immune Response in COVID-19

The authors set out to measure the mortality rate in COVID-19 attributable to excess immune response. Efforts were made to measure mortality due to the individual reported mechanisms.

Based on the articles included in the review, the deaths attributable to excess immune response and its individual mechanisms as reported above could not be quantified due to a paucity of data. Mortality due to severe respiratory failure was reported to be up to 60% [42]. In another study, respiratory failure was found to be the leading cause of COVID-19 death in 69.5% of patients [43]. These estimates are close to the findings of Wu et al., who had found the mortality due to ARDS to be 52.4% [44]. The mortality rate among patients with disseminated intravascular coagulation (DIC) in COVID-19 was reported to be 71.4% [45]. 

The overall case fatality rate of COVID-19 was reported to range from 1% to 10% [46]. The mortality rate in severe disease was reported to be 4% to 15% [47]. The case fatality rates for COVID-19 in various co-morbidities were documented to be 10.5% in patients with cardiovascular disease, 7.3% in patients with diabetes, and 6.0% in those with hypertension [8]. The case fatality rate in patients who did not have any co-morbidity varied from 3% to 4% [8]. Deaths in females ranged from 29% to 85% of total deaths, the latter being in older females [48]. While several lab investigations indicated the severity of disease, authors reported that a D-dimer level of more than 1 mg/liter predicted an 18-fold increase in the risk of death [49]. Another study reported a 10% increased risk of mortality with every 10% increase in the level of D-dimer or IL-6 [50].

### 3.3. Reporting of Mechanisms of Excess Immune Response

Since hyper-inflammation has been found predominantly in patients in severe COVID-19 [51], the mechanisms reported by the review have been observed primarily in severe COVID-19, unlike the initial stage of the infection. The mechanisms of excess immune response shown by the human immune system to SARS-CoV-2 were reported differently by various authors in the articles included in the review. A total of 14 different extracellular mechanisms were suggested by authors, while another four articles explained receptor-level activity. Another three articles exclusively explained intracellular mechanisms. Very few articles described intracellular continuum of extracellular mechanisms.

## 4. Discussion

We evaluated various mechanisms proposed or demonstrated by the authors in the articles included in the review. The same are discussed and summarized hereunder. We also tried to find out the common pathways among the reported mechanisms as discussed.

### 4.1. Mechanisms of Excess Immune Response of the Human Immune System due to SARS-CoV-2

#### 4.1.1. Extracellular Mechanisms

##### Cytokine Storm and Related Mechanisms

Cytokines are a family of small molecular proteins secreted by immune cells and tissues. These include IL, colony-stimulating factor (CSF), IFN, tumor necrosis factor (TNF), growth factor (GF), and chemokines [43]. Cytokines take part in immune responses through the activation of many signaling pathways like Janus kinase (JAK) signal transducer and activator of transcription protein (STAT), TNF receptor-associated factor (TRAF)-NF-κB, TRAF-activator protein 1 (AP-1), and interleukin-1 receptor-associated kinase (IRAK)-NF-κB [43]. The cytokine storm is described as a systemic acute inflammatory manifestation during viral infections in which the cytokine level rises in the body [46]. The cytokine storm observed in COVID-19 is more complex than other conditions due to the heterogeneous nature of hyper-inflammation at various stages of the disease [52] and due to a complex interaction of various components and axes [53]. It has been proposed to present in various forms such as Macrophage Activation Syndrome (MAS) or secondary haemophagocytic lymphohistiocytosis (sHLH), which are hyperferritinemic conditions [54]. The other mechanisms like immune-thrombosis or neutrophil extracellular traps (NET) are also associated with excess cytokine release. Delayed but elevated levels of pro-inflammatory cytokines and chemokines and delayed production of viro-protective IFNs lead to dysregulated immune response and cytokine storm with inefficient viral clearance [55]. 

##### Hyperferritinemia

Alunno et al. expressed that COVID-19 can be another hyperferritinemic condition like sHLH and MAS, which is characterized by persistent IFNγ-dependent stimulation of toll-like receptors (TLRs), antigen-presenting cells, and T-cell-uncontrolled activation, leading to cytokine storm [52]. The excess ferritin circulating in the body may originate either due to active secretion by macrophages and hepatocytes or due to the death of the cells. The circulating ferritin shows pro-inflammatory activity. Moreover, free iron released from ferritin leads to oxidative stress on red blood cells (RBC) and fibrin, which induces coagulation [56] and triggers multiple chain reactions [57]. RBCs are also damaged due to the tropism of the virus [58]. Damaged RBCs further release iron in circulation due to the disruption of hemoglobin [58]. Heavy-molecular-weight ferritin is shown to regulate an iron-independent signaling pathway, which eventually activates NF-κB [59] and causes secretion of excess cytokines. Removal of excess iron is done by the phagocytic system [60].

##### MAS and sHLH

Excessive cytokines result in activation of macrophages, which in turn increases the secretion of more cytokines like IL-6 and IL-10 [45] and contributes to lung damage [61]. McGonagle et al. elaborated that MAS in COVID-19 is atypical and that MAS-like lung inflammation and coagulopathy are more centered on the lung, which is aggravated by virus-induced immune suppression [62]. Galectin 3 is a carbohydrate-binding protein expressed by macrophages, epithelial cells, and alveolar cells, which drives macrophage-related hyper-inflammation, mediates viral adhesion, and promotes lung fibrosis [63]. Girija et al. proposed distinct mechanisms of three phases of cytokine storm [46]. Initial infiltration of the airway is induced by IFN-αβ and IFN-γ through mechanisms involving Fas–Fas ligand (FasL) or TNF-related apoptosis-inducing ligand (TRAIL)–death receptor 5 (DR5) [55]. This causes the apoptosis of airway and alveolar epithelial cells and damage to the pulmonary microvasculature, leading to vascular leakage, alveolar edema, and hypoxia [55]. In the next phase, TNF-mediated T-cell apoptosis occurs [46]. Activated macrophages accumulate in lung tissues through the abrogation of myeloid-specific STAT-1 signaling [46]. In the final phase, ARDS occurs due to IL-6, chemokine (C-X-C motif) ligand (CXCL)8, IL-1β, and Granulocyte-macrophage colony-stimulating factor (GM-CSF), chemokine (C-C motif) ligand (CCL) 2, CCL5, IFN-γ inducible protein (IP) -10, and CCL3 [46].

The role of IL-6 in the distinct MAS-like lung inflammation remained unclear to McGonagle et al. [62]. Another study demonstrated that lung inflammation and sustained cytokine production was due to the fact that IL-6 attenuates Human Leukocyte Antigen–DR isotype (HLA-DR) membrane expression in CD14 monocytes and decreases the production of IFN-γ by CD4 cells [42]. This leads to defective antigen presentation and lymphopenia, which causes the defective function of lymphoid cells [42,64], whereas monocytes remain potent and keep producing TNF-α and IL-6 [42].

The presentation of severe COVID-19 resembles sHLH, due to aberrant activation of T cells, NK cells, and macrophages, causing overproduction of inflammatory cytokines and hemophagocytosis [65].

##### Neutrophil-Related Mechanisms

Excess neutrophils sustain inflammation in COVID-19 [66]. Due to the need to produce more neutrophils, bone marrow can produce fewer cells of other types [67]. NETs are extracellular webs secreted by activated neutrophils under the influence of inflammatory cytokines to prevent the spread of pathogens and facilitate the accumulation of antimicrobial factors. The release of NETs is induced by damage-associated molecular patterns (DAMPs), particularly the high-mobility group box 1 (HMGB1) [68]. NETs are of two types: suicidal NETs and vital NETs. Suicidal NETs require the generation of reactive oxygen species (ROS) and activation of Raf/ mitogen-activated protein kinases ERK (MERK)/ extracellular signal-regulated kinases (ERK) pathway, while vital NETs sustain longer [68]. Laforge et al. postulated that excess ROS generated due to neutrophils leads to oxidative damage and excess immune response [69]. Inhibition of nuclear factor erythroid 2-related factor (NRF) 2-mediated pathways responsible for antioxidant defenses, and activation of NF-κB signaling can promote inflammation and oxidative damage during respiratory infections [69]. Small pathogens lead to excessive NETosis [68]. Excessive NET formation tends to aggravate secretion of more pro-inflammatory cytokines and formation of microvascular thrombosis by stimulating the intrinsic pathway through activation of Factor XII and propagating a pro-coagulant state [18,70]. NETosis in lung tissue also results in an insufficient anti-viral response by negatively regulating T cells and NK cells [47]. At high levels of HMGB1, pulmonary expression of the receptor for glycated end products (RAGE) is reported to cause detrimental inflammasome activation [71]. Misbalance in neutrophil serine cascade activator proteases and their inhibitors cause a proteolytic storm, which advances to a cytokine storm [72].

##### Immunothrombosis

Immunothrombosis or thromboinflammation is a process of formation of blood clots due to the interaction of platelets, coagulation factors, and innate immune effector systems like monocytes/macrophages, polymorphonuclear neutrophils, and the complement system [18]. COVID-19-induced coagulopathy results from platelet hyper-reactivity, hypercoagulability, hypo-fibrinolysis due to an imbalance between tissue plasminogen activator (tPA)/urokinase plasminogen activator (uPA) and plasminogen activator inhibitor-1 (PAI-1), complement overactivation, and renin–angiotensin aldosterone system (RAAS) derangement in the presence of underlying inflammatory-induced endothelial dysfunction [18]. Prolonged prothrombin time (PT) and minimally affected activated partial thromboplastin time (aPTT) in COVID-19 suggest a predominant tissue factor-factor VIIa (TF-F VIIa)-mediated activation of the extrinsic coagulation pathway. COVID-19-associated coagulopathy has been proposed to be pulmonary-specific intravascular coagulopathy or local DIC [73]. A bidirectional interaction between inflammation and coagulation has been observed. Innate immunity, pro-inflammatory cytokines, chemokines, adhesion molecules, tissue factor expression, platelet and endothelial activation, and micro-particles promote coagulation [73]. In turn, the activated coagulation products, including thrombin, Factor Xa, fibrin, and the TF–FVIIa complex through activating protease-activated receptors (PARs), induce secretion of pro-inflammatory cytokines [73]. Platelet-dependent protection of endothelial barrier integrity warrants differentiation of pro-thrombotic and pro-inflammatory mechanisms of platelets [74]. Anticoagulant protein S is encoded by PROS1 gene and is released due to rupture of the tissue containing it [49]. Lemke et al. hypothesized that excessive blood clotting and excess immune response are linked by consumption and exhaustion of protein S by the growing clot, which leads to inactivation of the immunosuppressive Mer receptor tyrosine kinase (MERTK) on macrophages and leads to the secretion of excess cytokines [49]. 

##### Other Mechanisms

Lymphocyte-Related Mechanisms

Song et al. demonstrated that despite a decrease in the absolute counts of CD8+ T cells, over-activation of these cells increased T-cell inhibitory molecules expression and increased multiple cytotoxic granules expression result in excess acute inflammation [75]. An increase in inhibitory receptors T-cell immunoglobulin mucin-3 (TIM-3) and Lymphocyte-activation gene-3 (LAG-3) on T effector cells modulates pro-inflammatory T cell responses [76]. Lymphopenia was found to be due to activation of apoptosis and the P53-signalling pathway in lymphocytes [77]. Exhaustion of lymphocytes causes the secretion of excessive inflammatory cytokines as a compensatory mechanism [54,78]. Compromised mechanisms of innate immune response pave the way for cytokine storm. Delayed response of type I IFNs (IFN α and β) hampers innate immune response against viruses through JAK-STAT signal transduction pathway. TLRs recruit signal transfer proteins and eventually activate the NF-κB pathway to secrete cytokines [79]. Cavalli et al. demonstrated predominant B-cell activation, which depends on a mammalian target of rapamycin (mTOR) pathway and can lead to hyper-inflammation [80]. A cross-referenced article also proposed antibody-dependent enhancement as a cause of cytokine storm [81]. 

Multiple Organ Damage

An et al. review described that pattern recognition receptors (PRR) recognize pathogen-associated molecular patterns (PAMP) during viral infections and activate IFN regulatory factor (IRF) and NF-κB, which promotes the release of pro-inflammatory cytokines and chemokines from infected local cells in the lungs [43]. The pro-inflammatory cytokines and chemokines are spilled over into the circulatory system, causing systemic cytokine storm and multiple organ damage [43]. Mechanisms of cardiac injury include direct myocardial injury by the virus through ACE2 entry, which is mediated by thrombaxane A2 [82], hypoxia-induced myocardial injury, microvascular damage and endothelial shedding, and cytokine/inflammation-mediated damage [8,83]. We support the view that the toxin effect of histamine on H1 and H2 receptors in myocardium causes cardiotoxicity [84]. Systemic hyper-inflammation leads to CNS complications [7]. Rare direct neuro-invasion causing CNS complications remains unproven to date [7]. Similarly, other organs also suffer damage due to hypoxia and deregulation of the control mechanism of inflammation [85]. 

A feature of COVID-19 is that the acute condition goes into chronic condition abruptly over a small period [86]. Due to the ongoing chronic inflammation, we are of the opinion that all organs predisposed to COVID-19-related damage will be affected even in the post-COVID phase. The post-COVID sequelae may include interstitial lung disease, dilated cardiomyopathy, chronic renal failure, cirrhosis of liver, other fibrotic conditions of organs, vasculopathies, psychiatric disorders, neuropathies, and arthropathies [87,88,89,90,91,92]. 

Hyper-inflammation in sub-groups of patients

NETs increase due to diabetes, obesity, increasing age, and male gender. All these conditions also show a shift from Th1 to Th2 cytokine response [68]. Testosterone has been found to induce DAMP release, resulting in increased TLR4 signaling in males [68]. Giaglis et al. proposed that Progesterone in females protects them from NETosis [93]. On the other hand, Rossi et al. proposed that estrogen in women stimulates cannabinoid receptor type 2, which leads to a limit of the release of pro-inflammatory cytokines, a shift of the macrophage phenotype towards the anti-inflammatory M2 type, and an enhancement of the immune-modulating properties of mesenchymal stromal cells [94]. O’Brien et al. proposed that the regulation of expression and extracellular release of Heat Shock Protein-HSP27 by estrogens is responsible for the relative protection of females from severe COVID-19 [48]. Blagosklonny et al. proposed that COVID-19 is an age-dependent syndrome associated with inflammaging and immunosenescence, hyperinflammation, hyperthrombosis, and cytokine storms as explained by the hyper-function theory of quasi-programmed aging [95].

#### 4.1.2. Intra-Cellular Mechanisms

It is proposed that rapid and excessive stimulation of the innate immune response triggers activation of the Nod-like receptor family, pyrin domain-containing 3 (NLRP3) inflammasome pathway in response to recognition of PAMPs or DAMPs like endogenous or exogenous adenosine triphosphate (ATP), ROS, or lysosomal proteases [48,96]. This activates PRRs such as TLRs, which bind to the virus [48], or nucleotide-binding oligomerization domain-containing protein 2 (NOD2). Eventually, NF-κB is activated, leading to the release of pro-inflammatory cytokines, causing acute lung injury [96]. NLRP3 activation leads to pyroptosis of infected cells, which is an inflammatory programmed cell death pathway of T lymphocytes mediated by IL-1β and IL-18 [96]. 

According to Saleh et al., the cytokine storm, oxidative stress, microbiota dysregulation, iron overload, and accumulation of ROS cause intra- and extra-mitochondrial dysfunction [50]. Dysfunction of the platelet mitochondria leads to coagulopathy [50].

#### 4.1.3. Signaling Pathways in an Excess Immune Response

Cytokine storm is characterized by ACE2 receptor-mediated inflammatory response, cell pyroptosis, delayed IFN α and β response by blocking STAT1 phosphorylation, and Anti S IgG-mediated lung injury [79]. The IL-6/JAK/STAT signaling pathway is reported to transduce extracellular signals transmitted by many pro-inflammatory factors [97]. Mahmudpour et al. explained that excess inflammatory cytokines are released due to down-regulation of ACE2 through dysregulation of the renin–angiotensin–aldosterone system (ACE/angiotensin II/angiotensin II type 1 receptor-AT1R axis) [53], which results in overproduction of angiotensin II, thereby enhancing IL-6 production via JAK/STAT pathway, and ultimately results in exacerbation of vascular and lung injuries [97]. Down-regulation of ACE2 leads to hyper-activation of NF-κB by IL-6 STATs axis; attenuation of Mas receptor (ACE2/MasR axis); production of Angiotensin-(1–7) that reduces the expression of p38 mitogen-activated protein kinase mitogen-activated protein kinase (MAPK) and NF-κB and inflammatory factors such as IL-6, TNFα, and IL-8; increased activation of [des-Arg9]-bradykinin (DABK) (ACE2/bradykinin B1R/DABK axis); and activation of the complement system including C5a and C5b-9 components [53]. Further, the angiotensin II/AT1 receptor axis activates a disintegrin and metalloproteinase (ADAM) 17, which cleaves and inactivates ACE2 and enhances angiotensin II retention [97]. For the complete induction of NF-κB pathway, the activation of STAT3 is reportedly required [97].

The sphingosine-1-phosphate (S1P)/sphingosine-1-phosphate receptor 1 (S1PR1) axis has been demonstrated to regulate the migration of numerous types of immune cells, including T and B lymphocytes, NK cells, and dendritic cells, and to inhibit the pathological damage induced by the host innate and adaptive immune responses [97].

The S1PR1 pathway agonism is documented to suppress cytokine and chemokine production, independently of TLR3 and TLR7 signaling or other endosome and cytosolic innate pathogen-sensing pathways, by targeting myeloid differentiation primary response gene 88 (MyD88)/TIR (Toll/interleukin-1 receptor)-domain-containing adapter-inducing IFN-β (TRIF) signaling, which are common actors with the NF-κB pathway [98].

NF-κB is a family of inducible transcription factors and a central mediator of induction of pro-inflammatory genes [17]. Exacerbation of NF-κB activation is implicated as the underlying mechanism in lung inflammatory pathology induced by respiratory viruses including SARS-CoV [97]. Moreover, SARS-CoV spike protein is documented to be associated with an increase in I-κBα degradation, which leads to activation of NF-κB pathway [97]. NF-κB levels were found to be higher in SARS-CoV-2-infected lungs, and suppression of this pathway enhanced IFN-mediated antiviral immunity and improved the infection outcome [99,100].

Based on the findings of the review, we observed a common pattern in the proposed mechanisms by various authors. Most of the described mechanisms interact bi-directionally with inflammatory cytokines by being induced by them and in return secreting more pro-inflammatory cytokines. Eventually, most mechanisms result in cytokine excess or storm. At the intra-cellular level, up-regulation of NF-κB was documented to play a major role in most of the mechanisms of excess immune response, as explained by the authors, including macrophage activation with release of cytokines, T-cell activation, and regulation of inflammasome particularly of NLRP3 variety [17]. However, we admit the limitation that not all studies explained the proposed mechanism up to the signaling at the intra-cellular level.

### 4.2. Role of Histamine and NF-κB in Excess Immune Response in COVID-19

**Figure 3:**
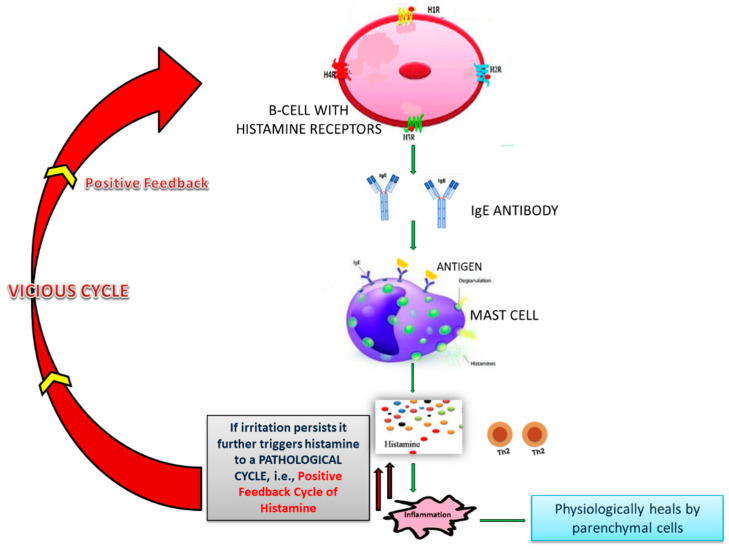
Positive feedback cycle of histamine.

Conti et al. showed that histamine increases IL-1 levels, causing hyper-inflammation in COVID-19 and cytokine storm [101]. Types I, II, and III reactions of the B-cell arm produce Th2 subset cytokines, while Th1 subset cytokines are produced by cell-mediated type IV reaction of T-cell arm [102,103,104,105,106,107,108]. The homeostasis between Th1 subset cytokines and Th2 subset cytokines is lost and excess Th2 cytokines arrest the activity of Th1 subset cytokines [109,110,111]. The Th2 subset cytokines give positive feedback to the IgE-producing B-cells and result in increased production of IgE [112,113,114,115,116]. Bax et al. showed that excess IgE causes excess histamine release from mast cells [111]. Any viral product remaining in the body can prolong the phase of fighting the insult and can exaggerate the process of inflammation [117,118]. Remnants of microorganisms like SARS-CoV-2 try to give an impression that there is still a persistent foreign particle [119,120]. Due to a prolonged fight against the insult, adrenaline gets exhausted and is unable to neutralize excess histamine, as the body has higher reserves of histamine since the mast cells are greater in number and are strategically placed all over the body. This un-neutralized histamine causes histamine imbalance and leads to the release of more pro-inflammatory Th2 cytokines [110,111,121,122]. In this way, an IgE-mediated positive feedback vicious cycle (Figure 3) is established, which results in excessive inflammation [109,110,111].

**Figure 4:**
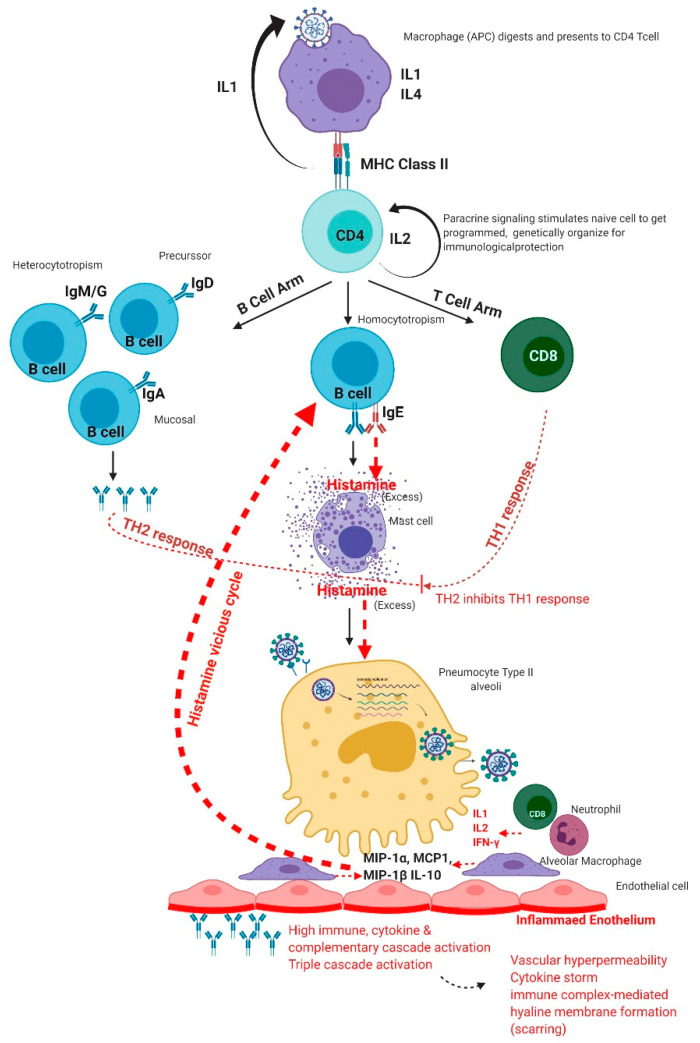
Excess immune response of the human immune system to SARS-CoV-2 infection.

Excess histamine causes excess diapedesis of all immune cells, antibodies, and mediators through vascular endothelium into the alveoli [6,23,123,124,125,126]. To prevent the excess diapedesis, a serous gel-like fluid is secreted at the site, which eventually thickens. This gel is made by three cascade reactions—complement, kinin, and coagulation. Ghebrehiwet et al. showed that the three cascades are linked with one another by Hageman factor (factor XII) [127], and when coagulation starts, all other cascades also start [127]. Complement cascade makes membrane attack complex (MAC) to kill the viruses [128]. Kinin pathway does the autonomic regulation [129]. Chaudhry and Babiker postulated that coagulation triggers the fibrinogen pathway and arrests the viruses from going anywhere [130]. Further, Nayak et al. showed that SARS-CoV-2 infects type II pneumocytes, leading to reduced production of surfactant (Figure 4) [131]. This causes a collapse of the alveoli, retention of fluid in lungs, and progressive hypoxemic respiratory failure [131,132].

Holden et al. showed that the dysregulated immune reaction is enhanced by excess histamine results due to the potentiation of NF-κB-dependent transcription and release of pro-inflammatory factors at the intracellular level [133]. Ayoub et al. explained that NF-κB exists in an inactive state in the cytoplasm of cells bound to its inhibitory protein I-κB. Phosphorylation of I-κB by IκB kinase (IKK) enzyme leads to its degradation. This results in translocation of NF-κB to the nucleus, where it promotes transcription of IL-10 and many pro-inflammatory factors like TNF-α, IL-1β, and IL-6 [134]. 

### 4.3. Drugs Acting on NF-κB Signaling

Various possible mechanisms of attenuation of hyper-inflammation include antagonism of IL6 receptors, inhibition of the JAK/STAT signaling pathway, agonism of S1PR_1_, blockade of TNFα, and down-regulation of NF- κB, either directly or indirectly by neutralization of excess histamine [97,133]. The findings of this review provide enough evidence of the benefit of targeting NF-κB down-regulation in the attenuation of the excess immune response to COVID-19. We researched various drugs and therapeutic approaches that can serve this purpose. NF-κB is required for normal immune response and survival of the cell. Hence, global inhibition of NF-κB signaling may affect the normal functioning of the cells due to the complexity of intrinsic pathways [17,97]. Liu et al. expressed that the development of drugs for selectively down-regulating NF-κB for clinical use has been a challenge for current science [17]. Various drugs have shown the ability to down-regulate NF-κB by acting upon different steps of its activity. Selective IKK inhibitors block the IKK-dependant phosphorylation of I-κB; proteasome inhibitors such as Bortezomib block I-κBα degradation, tacrolimus, and I-κBα super-repressor block nuclear translocation of NF-κB; and glucocorticoids and Peroxisome proliferator-activated receptors (PPAR) agonists block binding of NF-κB to deoxyribonucleic acid (DNA) [17]. Caffeic acid phenethyl ester (CAPE), Bay 11–7082, and parthenolide have been shown to inhibit NF-κB activation and reduce inflammation [97,99]. Hiscott et al. highlighted that molecules blocking NF-κB either are undergoing clinical trials or lack specificity and may cause unwanted side effects like broad suppression of innate immunity [135]. Hence, direct targeting of downstream effectors like TNFα by monoclonal antibodies like infliximab and adalimumab, which block TNFα, has been attempted to attenuate hyper-inflammation in several immune-mediated disorders [97]. These monoclonal antibodies are expensive, and drugs like adalimumab are under investigation in COVID-19 patients [136]. Galloway et al. reported that the major issue with TNFα-blockers has been an increased risk of bacterial and fungal superinfections [137], which can be a cause of concern in COVID-19 patients, who as documented by Zhou et al., mostly suffer superadded lung infections due to pre-existing foci [138]. Zhou et al. reported that at least one in seven COVID-19 patients encounter a secondary bacterial infection resulting in 50% of the fatalities due to untreated or untreatable secondary bacterial infections, occurring mostly in the lung [138].

Since histamine potentiates pro-inflammatory effects of NF-κB, targeting the upstream mediator histamine is a highly potential therapeutic approach in current times [22]. This approach not only helps in down-regulating NF-κB effectively and safely but also helps in reducing other effects of excess histamine. 

### 4.4. Neutralization of Excess Histamine and Down-Regulation of NF-κB

The need to neutralize histamine by some exogenous agent to prevent excess immune response was perceived by researchers in the past for various diseases. One approach, which is widely used in clinical practice, is the use of anti-histaminic drugs, which work by attaching to histamine receptors, but the persistent histamine molecules in the milieu behave like a toxin and cause toxin-mediated damage as reported by Comas-Basté et al. [139]. Moreover, histamine receptors are widely expressed in the body and are different in distribution between genders and age groups. This warrants caution in blocking histamine receptors and requires using a mixture of agonists and antagonists to avoid deleterious side effects [22]. Another approach based on the discussion presented above is the administration of exogenous adrenaline, but it cannot be tolerated by the body beyond a limit.

In 1951, Parrot and Laborde developed a new treatment method of co-administration of histamine and human serum gamma-globulin to restore the histamine-neutralizing ability of the body (histaminopexy). Immunoglobulin provides a larger co-molecule, and the complex of both the drugs behaves as a foreign body to produce antibodies within the body [140,141].

Haruo Yoshii and Yuriko Fukata administered intramuscular Histamine-conjugated-normal human immunoglobulin (Histamine dihydrochloride + Human normal immunoglobulin) for producing antihistamine antibodies within the human body [142,143,144,145]. This formulation reaches the lymph nodes and stimulates B cells to produce IgG antihistamine antibodies. The antibodies in turn reach the inflammatory site and neutralize the histamine directly, thereby enhancing the histamine-neutralizing ability of the plasma. 

The inventors recommended its use in the treatment of diseases associated with any abnormal immune response, including infectious diseases, parasitic diseases, respiratory diseases, and autoimmune diseases. A radioimmunoassay using a Histamine kit detected no residual histamine after administration of Histamine-conjugated-normal human immunoglobulin. An in vivo experiment in Bagg albino mice (BALB/c) for producing mouse-histamine-added gamma-globulins specific to trinitrophenyl showed significant promoting action upon IgG and IgM antibody production and potent immune-modulatory action. In an experimental allergic encephalomyelitis model of Female Lewis rats, the drug showed immune suppression comparative to cyclosporine A, proving the theory of positive feedback vicious cycle of hyper-inflammation due to histamine excess [142,143,144,145]. Administration of Histamine-conjugated-normal human immunoglobulin has shown remarkable clinical improvement in patients of diseases with an underlying excess immune response like urticarial, eosinophilia caused by a malignant tumor, chronic articular rheumatism, systemic lupus erythematosus, multiple sclerosis, and several diseases associated with hyper-eosinophilia [142,143,144,145,146]. 

Neutralization of histamine by Histamine-conjugated-normal human immunoglobulin has been demonstrated to inhibit the nuclear translocation of NF-κB and release of pro-inflammatory cytokines IL-1β, TNF-α, IL-6, and IL-10 [134]. The NF-kB independent effect of Histamine-conjugated-normal human immunoglobulin includes neutralization of histamine to reduce its toxin-like effects. Thus, Histamine-conjugated-normal human immunoglobulin has a great potential to reduce the excess immune response or hyper-inflammation encountered in diseases like COVID-19 without affecting the ability of the body to clear the virus, especially while specific and selective drugs for down-regulation of NF-κB are not available in current clinical practice.

In the wake of the COVID-19 pandemic, as a new drug takes around 12 to 15 years from its discovery to its use in patients, the only option left with the researchers and clinicians is to repurpose the existing therapeutics for use in COVID-19 [147]. In order to figure out which candidate molecules have a therapeutic effect against SARS-COV-2 and are suitable for repositioning studies, researchers have deployed various approaches to screen commercially available drugs. A few of these approaches are Computer-Aided Drug Design (CADD), molecular docking, re-trained multi-task deep model, homology modeling, virtual high-throughput drug screening, drug-likeness profiling, and docking scores [148,149,150,151,152,153,154]. Most of the approaches tried by other researchers are not based on human biology and the actual interaction of the virus with the human body. Hence, the drugs identified in such ways may not provide the expected therapeutic effect in clinical trials, which may cost lives due to delay in discovering ideal therapeutic candidates. The evidence generated by this review is based on the immunological mechanism of excess immune response in COVID-19 patients and can be useful in conducting further research and formulating guidelines for clinical practice.

## 5. Translational Value and Future Research Direction

To our best knowledge, this review is a state-of-art explanation of varied mechanisms of excess immune response in COVID-19, and it successfully brings out the common pattern of “cytokine excess and dysregulated cytokine response” among them. This review can form the basis for further research to understand the complex nature of hyper-inflammation in COVID-19 and can save significant suffering and loss of human life resulting from it. The review also critically appraises the available drugs for down-regulating NF-κB. Our approach of basing our proposition on real biological findings instead of computer-based modeling can provide a more real-world solution for the human body to cope with SARS-CoV-2 infection. The therapeutic candidate suggested by the review needs validation in clinical trials in COVID-19 patients.

## 6. Conclusions

The immunological findings in COVID-19 patients indicate that hyper-inflammation is the main culprit of morbidity and mortality in COVID-19. Histamine-conjugated-normal human immunoglobulin has been proved to be effective and safe in such situations based on the past data, due to its effect on down-regulation of NF-κB without directly blocking the NF-κB signaling pathway. It is already approved for use in humans and is available in the market for the last 30 years as an orphan drug for allergies. From an economic point of view, this treatment is inexpensive as compared to the methods currently used. The authors recommend clinical trials and further analysis of available data for repositioning of Histamine-conjugated-normal human immunoglobulin in COVID-19 for saving lives of the patients eliciting excess immune response.

## Figures and Tables

**Table 1:** Characteristics of the studies included in the review.

Characteristic ^1^	Number of Studies
Month of publication	N = 42
April 2020	6
May 2020	8
June 2020	12
July 2020	8
August 2020	3
September 2020	3
October 2020	2
Article type	N = 42
Research Paper	8
Systematic Review	1
Narrative Review	22
Editorial	1
Commentary	2
Comment	3
Hypothesis	2
Viewpoint/perspective	2
Updating article	1
Outcome (Mechanism of excess immune response)	N = 42
Extracellular mechanisms ^2^	
Cytokine storm	12
Neutrophil-related mechanism (NETosis, neutrophilia, and HMGB1 induced inflammation)	6
Lymphocyte-related mechanisms	4
Secondary haemophagocytic lymphohistocytosis	3
Direct injury	3
Immunothrombosis-related mechanisms	3
Hyperferritinemia	2
Macrophage activation syndrome (including Galactin-3 up-regulation)	2
Hypoxia-induced dysregulated immune response	1
Cannabinoid receptor-mediated immune suppression	1
Quasi-programmed aging	1
IL-6 attenuated HLA-DR expression	1
Intracellular mechanisms	
NLRP3 inflammasome activation	2
Dysfunction of platelet mitochondria	1
PROS1 signalling	1
NF-κB pathway	1
ACE2/bradykinin B1R/DABK axis involvement	1

^1^ All included articles were published in the English language and pertained to adult patients with severe COVID-19 with hyper-inflammation. ^2^ Two studies described both cytokine storm and direct invasion, while one study described both cytokine storm and secondary haemophagocytic lymphohistocytosis. Abbreviations: ACE2—angiotensin-converting enzyme 2; DABK—[des-Arg9]-bradykinin; HLA-DR—Human Leukocyte Antigen–DR isotype; HMGB1—high-mobility group box 1; NET—neutrophil extracellular traps; NF-κB—Nuclear Factor-κB; NLRP3—Nod-like receptor family, pyrin domain-containing 3.

## Data Availability

The data presented in this study are openly available in Harvard Dataverse at https://doi.org/10.7910/DVN/EQXE0E (accessed on 1 January 2021), reference number UNF:6:0kKzeLUxwDWCaZfoIrPIkg = = [fileUNF] [155].

## Pre-Registration

The review was prospectively registered in PROSPERO register with registration number CRD42020214230.
